# Self-Healing in Mobility-Restricted Conditions Maintaining Mechanical Robustness: Furan–Maleimide Diels–Alder Cycloadditions in Polymer Networks for Ambient Applications

**DOI:** 10.3390/polym12112543

**Published:** 2020-10-30

**Authors:** Dorothee Ehrhardt, Jessica Mangialetto, Jolien Bertouille, Kurt Van Durme, Bruno Van Mele, Niko Van den Brande

**Affiliations:** 1 Physical Chemistry and Polymer Science (FYSC), Vrije Universiteit Brussel (VUB), Pleinlaan 2, 1050 Brussels, Belgium; Dorothee.Ehrhardt@vub.be (D.E.); Kurt.Durme-van@dsm.com (K.V.D.); Bruno.Van.Mele@vub.be (B.V.M.); Niko.Van.den.Brande@vub.be (N.V.d.B.); 2 Organic Chemistry (ORGC), Vrije Universiteit Brussel (VUB), Pleinlaan 2, 1050 Brussels, Belgium; Jolien.Bertouille@vub.be; 3 DSM Advanced Solar, Urmonderbaan 22, 6167 RD Geleen, The Netherlands

**Keywords:** dynamic covalent bonds, reversible thermosets, kinetic simulations, self-healing in vitrified state, self-repair of microcracks, encapsulant for photovoltaics

## Abstract

Two reversible polymer networks, based on Diels–Alder cycloadditions, are selected to discuss the opportunities of mobility-controlled self-healing in ambient conditions for which information is lacking in literature. The main methods for this study are (modulated temperature) differential scanning calorimetry, microcalorimetry, dynamic rheometry, dynamic mechanical analysis, and kinetic simulations. The reversible network 3M-3F630 is chosen to study the conceptual aspects of diffusion-controlled Diels–Alder reactions from 20 to 65 °C. Network formation by gelation is proven and above 30 °C gelled glasses are formed, while cure below 30 °C gives ungelled glasses. The slow progress of Diels–Alder reactions in mobility-restricted conditions is proven by the further increase of the system’s glass transition temperature by 24 °C beyond the cure temperature of 20 °C. These findings are employed in the reversible network 3M-F375PMA, which is UV-polymerized, starting from a Diels–Alder methacrylate pre-polymer. Self-healing of microcracks in diffusion-controlled conditions is demonstrated at 20 °C. De-gelation measurements show the structural integrity of both networks up to at least 150 °C. Moreover, mechanical robustness in 3M-F375PMA is maintained by the poly(methacrylate) chains to at least 120 °C. The self-healing capacity is simulated in an ambient temperature window between −40 and 85 °C, supporting its applicability as self-healing encapsulant in photovoltaics.

## 1. Introduction

Self-healing polymer networks have the ability to heal (micro-)defects in order to maintain and restore functional properties [1,2,3,4,5,6]. The first stage of the healing process is the sealing step, in which the gap between crack surfaces is closed. This requires the creation of a sufficiently mobile phase, enabling close contact between both sides of the damaged site. The second stage is the healing step, during which the initial polymer properties are restored [4,7]. Typically, self-healing polymers are designed based on (i) an extrinsic approach using pre-embedded healing agents (e.g., microcapsules [8,9,10] or microvascular systems [11,12,13,14]) or (ii) an intrinsic approach using reversible covalent [15,16,17,18,19,20,21,22,23,24] or supramolecular [25,26,27,28,29] bonds.

**Figure 1:**
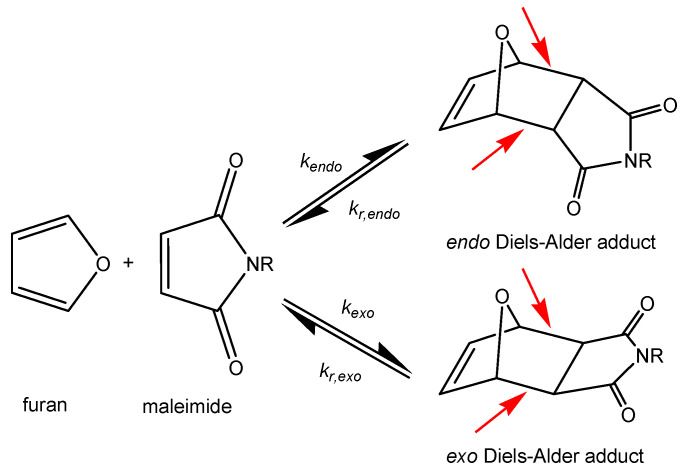
Reversible Diels–Alder reactions between maleimide and furan derivatives, forming *endo* and *exo* Diels–Alder adducts. Reversible Diels–Alder bonds are indicated by red arrows.

The thermoreversible Diels–Alder reaction between furan (a conjugated diene) and maleimide (a dienophile) is particularly interesting for the design of dynamic covalent self-healing polymer networks, because the equilibrium reactions can be repeated multiple times without side reactions, and the reaction kinetics are well studied [30,31,32,33,34,35,36,37,38,39]. Recently, the kinetics and equilibrium thermodynamics for polymer network systems formed from amorphous furan and maleimide have been updated to match a broader range of building blocks [40]. At low temperatures, the forward Diels–Alder reaction is favored, and Diels–Alder conversion increases. During this [4+2]-cycloaddition, the Diels–Alder adduct with two new stereogenic centers is formed. The relative orientation of the substituents in the Diels–Alder adduct is determined by two suprafacial approaches (*endo* and *exo*), leading to the formation of *endo* and *exo* Diels–Alder adducts (Figure 1).

The *endo* Diels–Alder adduct is kinetically favored, while the *exo* Diels–Alder adduct is thermodynamically more stable [41]. At high temperatures, the reaction equilibrium is shifted, and cycloreversion (retro Diels–Alder reaction) is favored. During retro Diels–Alder reaction, furan and maleimide are formed again by breaking Diels–Alder bonds. This causes the Diels–Alder conversion to decrease. Furthermore, Diels–Alder bonds possess a lower bond energy than other carbon-carbon σ-bonds [34,37,42], which means that these bonds are preferentially broken in case the material is damaged (Figure 1). Subsequently, the reversible nature of the Diels–Alder reaction allows Diels–Alder bonds to re-form, enabling repeatable healing of the material [33,34,36,39].

The majority of self-healing studies on reversible covalent polymer networks focusses on materials in the elastomeric state. This means that the sealing and healing stages are performed at temperatures (*T*_app_) higher than the material’s glass transition temperature (*T*_g_), at which the polymer network exhibits sufficient segmental mobility (*T*_app_ > *T*_g_) [34,36,43,44,45].

However, few studies can be found on self-healing polymer networks at temperatures below the *T*_g_, i.e., in the (partially) vitrified state (*T*_app_ < *T*_g_), even though it is very relevant in many applications (e.g., as self-healing encapsulant in photovoltaics or protective polymer network coatings in general) [38,39]. Here, reaction rates are diffusion-controlled, and thus, reduced due to mobility restrictions.

For fully reversible polymer networks, undesired loss of mechanical robustness and even loss of structural/geometrical integrity can be an issue in the sealing step at elevated temperatures. Here, crosslink density decreases to zero if the Diels–Alder conversion decreases below gel conversion (*x*_gel_) and de-gelation occurs above *T*_de-gel_, leading to unwanted changes in mechanical properties, such as a decrease in the elastic modulus to an insufficient level and eventually viscous flow [30,34]. However, sufficient mechanical robustness can be maintained over a broad temperature range, even below *x*_gel_, through (i) the use of interpenetrating polymer networks (IPNs), which consist of an irreversible polymer network of high(er) *T*_g_ in combination with a reversible polymer network of low *T*_g_ [46] (pp. 155–180), or (ii) by using a polymer network containing reversible crosslinks (e.g., Diels–Alder bonds) between irreversible polymer chains (e.g., poly(methacrylate) chains) [39].

In this work, two reversible polymer networks 3M-3F630 and 3M-F375PMA are selected to study network formation in diffusion-controlled reaction conditions and the self-healing potential at ambient temperature in the (partially) vitrified state. These research topics are innovative, both for the underlying theoretical aspects as well as for applications in ambient conditions, and are lacking in the literature of self-healing polymer materials. The system 3M-3F630 is based on a tris-functional maleimide (average functionality *f* = 2.65) and a tris-functional furan compound (functionality *g* = 3). The system 3M-F375PMA is synthesized from the same tris-functional maleimide (*f* = 2.65) and a mono-furan-mono-methacrylate compound (obtaining 3M-F375MA), followed by UV-polymerization (obtaining 3M-F375PMA). Both polymer networks are fully reversible as all crosslinks are reversible covalent Diels–Alder bonds based on furan and maleimide derivatives. The system 3M-3F630 is chosen to systematically study the influence of cure temperature on gelation and vitrification at different cure temperatures up to 85 °C as well as the de-gelation of the formed reversible network at more elevated temperatures. The progress of cure in the vitrified state at 20 °C is followed based on the evolution of *T*_g_ during cure. The continuation of Diels–Alder reactions under these conditions is an important prerequisite for self-healing in the vitrified state. These findings are exploited in the second system 3M-F375PMA. Self-healing of microcracks in diffusion-controlled conditions is demonstrated. It is shown that this fully reversible polymer network maintains mechanical robustness over a broad temperature range (due to the irreversible poly(methacrylate) chains). The simulated self-healing capacity in a more extended ambient temperature window between −40 and 85 °C for outdoor applications, and preliminary UV-Vis transmittance results support its potential applicability as self-healing encapsulant in photovoltaics.

## 2. Materials and Methods

### 2.1. Materials

**Figure 2:**
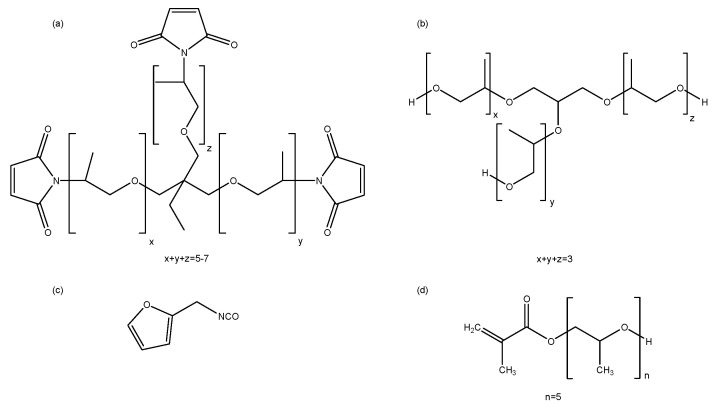
Chemical structures of the starting reagents: (**a**) amorphous trismaleimide 3M, (**b**) glycerol-based polyol Daltolac R630, (**c**) furfuryl isocyanate, (**d**) poly(propylene glycol) methacrylate PPG375-MA.

Amorphous propylene oxide trismaleimide (3M, *M*_eq_ = 249 g mol^−1^, *f* = 2.65) is purchased from Specific Polymers. Glycerol-based polyol Daltolac R630 (*M* = 267 g mol^−1^) is supplied by Huntsman. Poly(propylene glycol) methacrylate (PPG375-MA, *M* = 375 g mol^−1^, 97%), furfuryl isocyanate (*M* = 123 g mol^−1^), dibutyl tin dilaurate (DBTDL, catalyst) and phenylbis(2,4,6-trimethylbenzoyl) phosphine oxide or bisacyl phosphine oxide (BAPO, 97%, powder, photoinitiator) are purchased from Sigma Aldrich. Daltolac R630 is dried over molecular sieves (4 Å), all other materials are used as received. 3M is stored at 4 °C under inert gas atmosphere. Furfuryl isocyanate is stored at 4 °C. Chemical structures of all products are shown in Figure 2.

### 2.2. Synthesis

**Synthesis of trisfuran 3F630.** Equimolar amounts of Daltolac R630 and furfuryl isocyanate are allowed to react at ambient temperature, with DBTDL as catalyst. The reaction is considered complete, when the asymmetric stretching vibration of the isocyanate group υ_as_(N=C=O) cannot be detected anymore by means of attenuated total reflectance Fourier transform infrared (ATR FTIR) spectroscopy.

**Synthesis of furan-functionalized methacrylate F375MA.** The photoinitiator BAPO is dissolved in furfuryl isocyanate, and subsequently, an equimolar amount of PPG375-MA is added. The final BAPO concentration in F375MA is 0.1 wt%. The compounds are reacted in the presence of a catalytic amount of DBTDL, and the reaction progress is monitored by means of ATR FTIR as described above.

**Figure 3:**
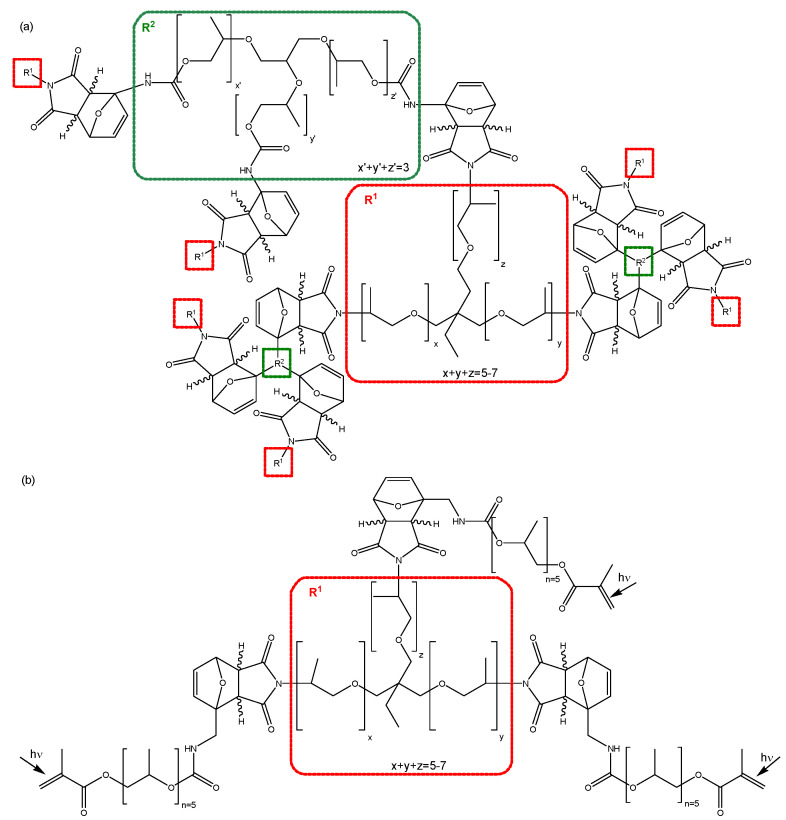
Chemical structures of the polymer networks (**a**) 3M-3F630 and (**b**) 3M-F375PMA.

**Synthesis of the reversible polymer network 3M-3F630.** Equimolar amounts of 3M and 3F630 (maleimide:furan = 1:1) are mixed at ambient temperature, and immediately transferred to the respective equipment for analysis. The reversible network structure is shown in Figure 3a.

**Synthesis of the reversible polymer network 3M-F375PMA.** The synthesis of 3M-F375PMA is a two-step procedure. In the first step, 3M and F375MA are mixed in equimolar amounts (maleimide:furan = 1:1) and allowed to react at ambient temperature for at least four days to form 3M-F375MA, a tris-methacrylate-functionalized reversible prepolymer containing Diels–Alder bonds. In the second step, 3M-F375MA is spread onto a glass substrate (treated with release agent) at ambient temperature. Subsequently, the sample is UV-polymerized for 30 min at 60 °C in nitrogen atmosphere with a UV-light intensity of 115 mW cm^−2^ (λ_max_ = 360 nm) to form polymer films 3M-F375PMA with a thickness of 300–500 µm. The network structure is shown in Figure 3b. For further details on the reaction procedure, see [39].

### 2.3. Methods

Fourier-transform infrared (FTIR) spectroscopy is performed on a Thermo Scientific Nicolet 6700 FTIR spectrometer. Spectra are recorded at ambient temperature between 4000 and 400 cm^−1^, with a resolution of 4 cm^−1^. All spectra are averaged from 32 scans. The progress of urethane reactions is monitored by means of ATR FTIR spectroscopy using a single reflection Smart iTR ATR sampling accessory with a zinc selenide (ZnSe) crystal. The urethane reaction is considered complete when the asymmetric stretching vibration of the isocyanate group υ_as_(N=C=O) at about 2250 cm^−1^ cannot be detected anymore. Reversibility of the UV-cured polymer network 3M-F375PMA is studied using transmission FTIR spectroscopy in potassium bromide (KBr) pellets with an approximate sample concentration of 1 wt%.

(Modulated temperature) Differential scanning calorimetry ((MT)DSC) is performed using a Discovery DSC 250 from TA Instruments equipped with a refrigerated cooling system. All experiments are performed under nitrogen atmosphere with samples of ca. 5 to 10 mg. Freshly prepared Diels–Alder mixtures are immediately measured or stored in liquid nitrogen to avoid conversion change. Hermetic *T*_zero_ aluminum pans with perforated lids are used to assure nitrogen purging in the DSC cell. Quasi-isothermal MTDSC experiments are carried out with a temperature modulation of 0.5 °C and a period of 40 s to follow the heat capacity change during reaction. Non-isothermal DSC measurements are performed at a heating rate of 5 K min^−1^ and at a cooling rate of 20 K min^−1^ to limit reaction during the cooling step, except stated otherwise.

Dynamic rheometry is carried out with a TA Instruments Discovery HR-2 hybrid rheometer, equipped with an environmental test chamber. All experiments are carried out between parallel plates of 10 mm diameter, using 0.3, 0.6, 1.0, 1.7 and 3.1 Hz as modulation frequencies. The gelation time *t_gel_* during an isothermal cure is defined as the time at which the loss angle becomes independent of frequency. Non-isothermal de-gelation measurements are performed with a heating rate of 0.5 K min^−1^.

Microcalorimetry is performed on a TA Instruments Thermal Activity Monitor (TAM III) in isothermal conditions at 20 °C (±0.1 °C). The equipment has a precision of ±200 nW and a baseline drift of maximum 200 nW in 24 h. For experiments regarding vitrification through Diels–Alder reaction, furan and maleimide compounds are mixed in equimolar amounts in a measuring ampoule, and the microcalorimetry experiment is started immediately. For self-healing experiments on UV-cured polymer networks, freshly prepared powder is compressed for 10 min at elevated pressure, then the compressed powder is inserted into a measuring ampoule, and the measurement is started without delay. Additionally, the residual heat flow of unground bulk material, which serves as reference, is measured via the same procedure.

Dynamic mechanical analysis (DMA) is carried out on a TA Instruments DMA Q800, using a gas cooling accessory. All experiments are performed at a frequency of 1 Hz between −40 and 120 °C, with a heating/cooling rate of 0.5 K min^−1^. UV-cured polymer network films are measured in tension mode, while powder rectangular bar samples are measured in 3-point bending mode. Powder rectangular bars are prepared by compressing freshly prepared polymer network powder for 10 min at elevated pressure in a mold with the dimensions 30 mm × 5 mm × 2 mm. Reference samples are immediately demolded and analyzed in DMA, whereas self-healed rectangular bars are allowed to heal for one week at 20 °C and 1 bar before being tested in DMA.

UV-Vis spectroscopy is performed on a PerkinElmer LAMBDA 35 UV/Vis spectrophotometer between 1000 and 200 nm at a scanning speed of 480 nm min^−1^ on UV-cured 3M-F375PMA polymer network films with an approximate thickness of 500 μm.

Kinetic simulations are performed using an in-house MATLAB software in which the equilibrium reactions between the Diels–Alder functional groups, furan and maleimide, forming *endo* and *exo* Diels–Alder cycloadducts are described by a mechanistic model shown in Figure 1 [34]. For both stereoisomers, the kinetics of the Diels–Alder and retro Diels–Alder reactions are described by their corresponding rate constants *k(T)*. The following set of differential equations is derived from this model:

(1) $$\frac{{dC}_{i}}{dt}=\overset{R}{\underset{j=1}{\sum }}{(v}_{i}^{j})$$

with *C_i_* (mol kg^−1^) the concentration and *dC_i_/dt* (mol kg^−1^ s^−1^) the production rate of component *i*, *R* the number of reactions involved (*R* = 2 or 4), $v_{i}^{j}$ (mol kg^−1^ s^−1^) the formation (or consumption) rate of component *i* in reaction *j*, which depends on the rate constant *k*_j_*(T)* and the concentrations of the components involved in reaction *j*. The differential equations can be related to the measured reaction heat flow profiles normalized against the sample weight:

(2) $$\frac{{dq}_{r}}{dt}=\overset{N}{\underset{i=1}{\sum }}\left[\frac{{dC}_{i}}{dt}{∆}_{f}H_{i}^{0}\right]$$

with *dq_r_/dt* (W kg^−1^) the experimental microcalorimetric heat flow, ${∆}_{f}H_{i}^{0}$ the formation enthalpy of component *i* (kJ mol^−1^) and *N* the number of components (*N* = 4).

The model parameters comprise the kinetic and thermodynamic parameters of the two Diels–Alder equilibria of Figure 1, and the functionalities of the furan and maleimide compounds. Parameter values optimized for Diels–Alder systems based on different combinations of furan-functionalized polyether amines (Jeffamines) with amorphous maleimides are used for the simulations of the two Diels–Alder systems of this paper ([40], see Table S1 in the Supplementary Materials). A representative reversible network system M400-3F251 (see Figure S1 in the Supplementary Materials), showing fully kinetically-controlled cure at 20 °C, is used to prove the validity of the data of Table S1 for all kinetic simulations in this paper (see Figure S2 in the Supplementary Materials).

For all simulations, the concentration of the starting products based on the starting weight fraction (i.e., [*F*]_0_ for the furan compound, [*M*]_0_ for the maleimide compound, [*Endo*]_0_ or [*Exo*]_0_ for the Diels–Alder adducts) are taken into account, along with the preparation time and temperature. The total Diels–Alder conversion *x*, describing the formation of the *endo* and *exo* adducts for each combination of time and temperature, is calculated according to:

(3) $${x=x}_{endo}+x_{exo}=\frac{\left[Endo\right]+\left[Exo\right]}{\min\left({\left[F\right]}_{0},\;{\left[M\right]}_{0}\right)+{\left[Endo\right]}_{0}+{\left[Exo\right]}_{0}}$$

with min([*F*]_0_, [*M*]_0_) the minimum value between [*F*]_0_ and [*M*]_0_ while the equilibrium conversion is based on:

(4) $$x_{eq}=\frac{{2K}_{tot}{\left[F\right]}_{0}+1-\sqrt{{\left({2K}_{tot}{\left[F\right]}_{0}+1\right)}^{2}{-4K}_{tot}^{2}{\left[F\right]}_{0}^{2}}}{{2K}_{tot}{\left[F\right]}_{0}}$$

with *K*_tot_ = *K*_endo_ + *K*_exo_.

## 3. Results and Discussion

### 3.1. Vitrification and Gelation during Cure of Reversible Network 3M-3F630 in Ambient Conditions

#### 3.1.1. Vitrification Study Based on Modulated Temperature Differential Scanning Calorimetry

During the cure of an irreversible thermosetting network at a temperature *T*_cure_ below the glass transition temperature of the full-cured network *T*_g1_ (*T*_cure_ < *T*_g1_), the *T*_g_ of the curing network progressively increases until it reaches the applied cure temperature (*T*_g_ ≈ *T*_cure_) where decreasing segmental mobility leads to vitrification and a subsequent slowing down of the reaction. An interesting technique to study this phenomenon is MTDSC. With this tool, the heat flow and heat capacity evolutions during reaction can be measured simultaneously, which give indications on the decrease in reaction rate during vitrification. This was proven in previous publications for irreversible thermosets [47,48,49] and recently also reversible furan–maleimide Diels–Alder thermosets [38]. A drop in heat capacity (Δ*c_p_*) shows the occurrence of the vitrification process as it expresses the progressive decrease of segmental chain mobility due to diffusion-controlled reactions (see [38] and [40] for more details).

In this paper, the same methodology is applied for the 3M-3F630 thermosetting system of maleimide functionality *f* = 2.65 and furan functionality *g* = 3 (see Section 2. Materials and Methods).

**Figure 4:**
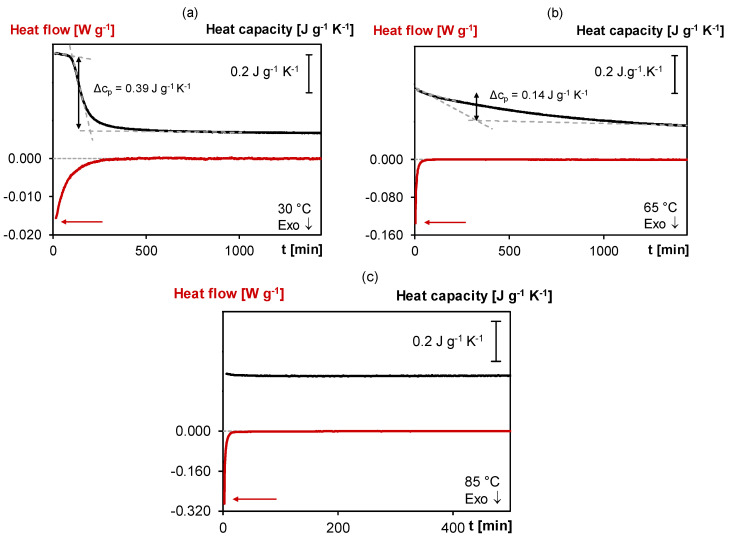
MTDSC thermogram of 3M-3F630 at isothermal cure temperatures of (**a**) 30 °C, (**b**) 65 °C, (**c**) 85 °C. **Black**: (specific) heat capacity as a function of time; **Red**: (non-reversing) normalized heat flow as a function of time.

Figure 4 shows the normalized non-reversing heat flow and specific heat capacity measured in quasi-isothermal MTDSC experiments of fresh 3M-3F630 mixtures cured for one day at 30, 65 and 85 °C. Here, the exothermic heat flow shows a maximum intensity at the beginning of the reaction, i.e., 16 mW g^−1^ at 30 °C, 135 mW g^−1^ at 65 °C and 290 mW g^−1^ at 85 °C, and is progressively decreasing over time. As observed, the forward Diels–Alder reaction rate is thus the highest at the beginning of the cure for the highest cure temperature. Subsequently, depending on the cure temperature, a sudden drop of *c_p_* can be observed, indicative for the first stage (onset) of the vitrification process. At 30 °C, the sudden Δ*c_p_* drop of −0.39 J g^−1^ K^−1^ occurs after 103 min while at 65 °C, the *c_p_* drops more progressively and by only −0.14 J g^−1^ K^−1^, showing partial vitrification. At 85 °C, no drop in *c_p_* is observed which shows that vitrification no longer occurs. Here, the higher the cure temperature, the lower will be Δ*c_p_* linked to the vitrification phenomenon, until no vitrification is visible anymore. These MTDSC measurements correlate with *T*_g_s of 50, 62 and 59 °C after quasi-isothermal cure at 30, 65 and 85 °C, respectively (*T*_g_s measured in a subsequent cooling at 20 K min^−1^ and heating step at 5 K min^−1^ in standard DSC mode). Note that the retro Diels–Alder reactions are more important at 85 °C than at 65 °C which explains the lower *T*_g_ at 85 °C than at 65 °C. After the initial vitrification step, the *c_p_* continues to slightly decrease over time for 30 and 65°C, showing that the reaction still proceeds even in the vitrified state for at least one day. This effect will be further explored in Section 3.2.2. A major benefit of MTDSC is the reliable detection of very small quasi-isothermal *c_p_* changes with high sensitivity, free of noise and not affected by a heat flow baseline drift. On the contrary, the baseline stability of the non-reversing heat flow of quasi-isothermal MTDSC measurements over a long cure time is insufficient to detect the weak exothermicity of a still ongoing reaction. For this purpose, microcalorimetry is more appropriate (see also Section 3.2.2.).

While the 3M-3F630 system can vitrify in ambient cure conditions around room temperature and even higher, this is not the case for the second reversible Diels–Alder system, the prepolymer 3M-F375MA, as shown in Figure S3 (in the Supporting Material) with a MTDSC *c_p_* and a microcalorimetric heat flow measurement at 20 °C. This is in agreement with the measured *T*_g_ of –31 °C after two days of cure. The prepolymer 3M-F375MA will only obtain thermosetting properties in ambient conditions after photo-cure into the reversible network 3M-F375PMA (see Section 2.2 Synthesis).

#### 3.1.2. Dynamic Rheometry as Proof of Gelation and Vitrification

**Figure 5:**
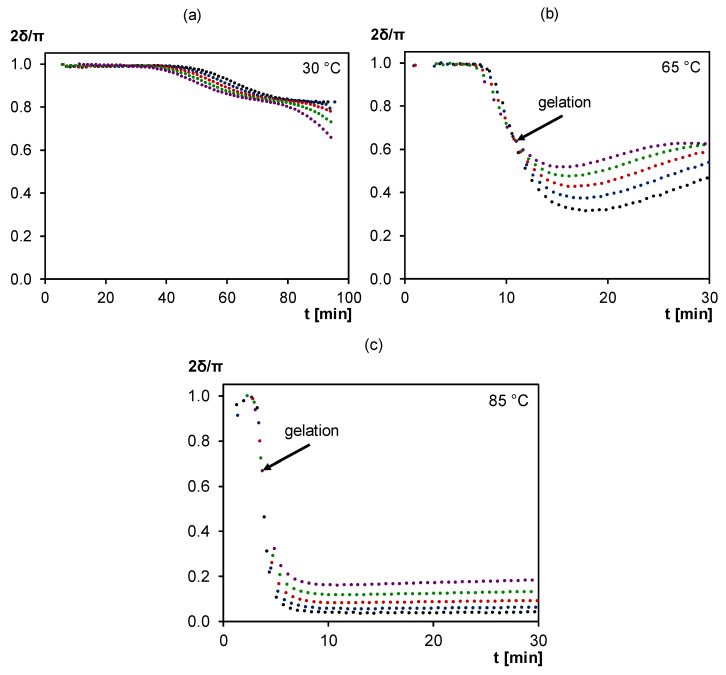
Normalized loss angle of dynamic rheometry during isothermal cure of 3M-3F630 at (**a**) 30°C, (**b**) 65°C, (**c**) 85°C. **Black**: 0.3 Hz; **Blue**: 0.6 Hz; **Red**: 1.0 Hz; **Green**: 1.8 Hz; **Purple**: 3.1 Hz.

In order to assure at least structural integrity of the material above *T*_g_, the polymer must have a network structure, preventing flow of the material (see also 1. Introduction). The formation of this network occurs during the gelation process. During cure, the conversion progressively increases until the gel point is reached, corresponding to the critical gel conversion *x*_gel_ at a certain time *t*_gel_, where an incipient network is formed (crosslink density is zero at this point). Afterwards, conversion further increases and crosslink density builds up until reaching an “arrested” equilibrium induced by vitrification (see Section 3.1.1). MTDSC is not suitable to characterize *t*_gel_, however, gelation can be studied by means of dynamic rheometry in isothermal conditions, as shown in Figure 5 for the 3M-3F630 system for cure at 30, 65 and 85°C.

Here, the frequency-independency of the rheometrical loss angle *δ* is chosen as criterion for the gel point determination as it is characteristic of the network formation for which the critical gel conversion *x*_gel_ is reached [50,51,52,53]. The crossing of *δ* is clearly observable at 65 and 85 °C (indicated in Figure 5 by arrows) but not at 30 °C. At 85 °C, the system behaves as an unvitrified thermoplastic until gelation after about 4 min. From then on, it shows elastomeric behavior as no vitrification is observed for the rest of the isothermal experiment (see also MTDSC at 85 °C in Section 3.1.1). At 65°C, the system behaves first as a thermoplastic until gelation after about 10 min. At this point, it shows elastomeric behavior until vitrification, observable through the following increase of *δ* due to the rising shear storage modulus (not shown) during (partial) vitrification. At 30 °C, no crossing of *δ* is observable which means that no gelation occurs before the start of vitrification. In this measurement, vitrification is observed with a frequency dependent peak in *δ* following its initial drop. Thus, at 30 °C, an ungelled glass is formed while at 65 °C, a gelled glass is obtained.

The critical gel conversion can be calculated using the preparation conditions, the rheometrical time-temperature program, the starting weight fractions of furan and maleimide compounds and the kinetic parameters from [40]. The calculated value is independent of cure temperature, as expected [40], and has a value of *x*_gel_ = 0.50. The critical loss angle at gelation for 3M-3F630 is between 60° and 65°, thus above 45°, meaning that gelation is still viscous-dominated [53]. This is also the case in other reversible Diels–Alder networks [34,40].

At 30 and 65°C, vitrification is observed at similar times as with MTDSC measurements (see Section 3.1.1). However, dynamic rheometry is less suited than MTDSC to describe the vitrification process in a quantitative way due to the fact that this measuring technique is designed for liquids and visco-elastic materials and not for thermosetting solids with too high elastic moduli.

Note that for 3M-F375MA gelation measurements are not meaningful as no reversible network is formed through the forward Diels–Alder reactions in the first step of the synthesis. The reversible network 3M-F375PMA is only formed during the second step of the synthesis by UV-cure (see Section 2.2 Synthesis for more details).

### 3.2. Proof of Progress of Cure in Diffusion-Controlled Conditions at 20 °C in Reversible Network 3M-3F630

#### 3.2.1. *T*_g_–*x* Relationship

**Figure 6:**
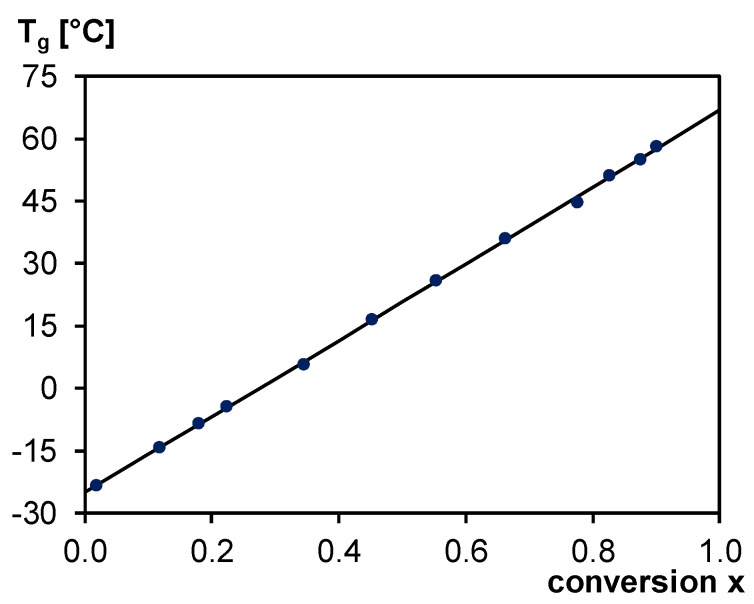
*T*_g_–*x* relation for 3M-3F630. **Black line**: DiBenedetto model with optimized *T*_g0_, *T*_g1_ and λ; **Blue dots**: experimental *T*_g_-calculated *x* couples.

The *T*_g_–*x* relation of a reversible network is an important tool to evaluate its structure-property relations. This *T*_g_–*x* relation allows the direct translation of a calculated conversion *x* in terms of *T*_g_. To build this relation, *T*_g_s of fresh mixtures are established by DSC for specific time-temperature programs that are simulated with the kinetics model in order to determine their corresponding conversion. A typical experiment consists of an isothermal segment for a well-chosen time-temperature combination at which reactions stay kinetically controlled, followed by a cooling at 20 K min^−1^ (to limit further reaction) and a heating step at 5 K min^−1^ (to measure the *T*_g_). The corresponding conversions *x* are calculated with the kinetics model considering the starting weight fractions and functionalities of furan and maleimide compounds, the preparation conditions, the DSC time-temperature program, and the kinetic parameters from [40]. As shown in Figure 6, the *T*_g_–*x* couples are often fitted to the empirical DiBenedetto model:

(5) $$\frac{T_{g}{-T}_{g0}}{T_{g1}{-T}_{g0}}=\frac{\lambda x}{1-\left(1-\lambda \right)x}$$

where λ represents a fitting parameter, *T*_g0_ is the glass transition temperature of the unreacted system (i.e., at *x* = 0) and *T*_g1_ the one corresponding to a fully cured system (i.e., at *x* = 1) [54,55,56].

Due to the minimum preparation time required, *T*_g0_ cannot be measured since a minimum conversion of around 0.02 is expected at room temperature. Likewise, *T*_g1_ cannot be measured either as full conversion in such Diels–Alder systems can only be reached at low cure temperatures where vitrification and diffusion-controlled reaction are unavoidable. Only *T*_g_s for a range of conversion between *x* = 0.02 and *x* = 0.90 could be experimentally measured.

The optimized parameters of the DiBenedetto model are −25°C for *T*_g0_, 67°C for *T*_g1_ and 0.99 for *λ*. The resulting *T*_g_–*x* curve is shown in Figure 6. Note that during the different experiments to construct the *T*_g_–*x*, between 30% and 92% of *exo* Diels–Alder adduct is formed. Nevertheless, a unique *T*_g_–*x* relation is found, linking *T*_g_ to the overall (*endo*+*exo*) Diels–Alder conversion and validating the assumption that the *T*_g_s of the *endo* and *exo* cycloadducts are (almost) equal.

#### 3.2.2. Cure in Diffusion-Controlled Conditions at 20 °C

**Figure 7:**
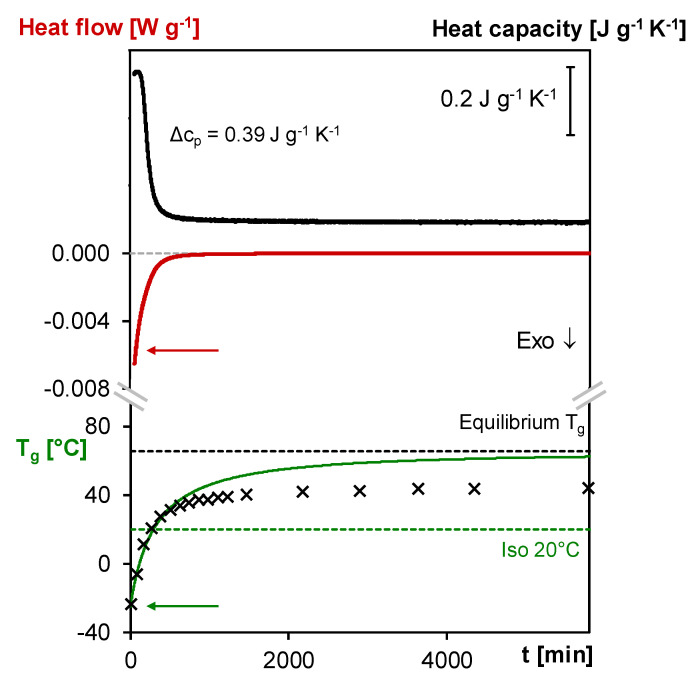
MTDSC, microcalorimetry, simulated and experimental evolution of the *T*_g_ as a function of time at 20 °C of the 3M- 3F630 system. **Full black line**: (specific) heat capacity measured in MTDSC; **Full red line**: heat flow measured in microcalorimetry; **Full green line**: simulated evolution of the *T*_g_; **Black crosses**: experimental *T*_g_ measured at different time of cure at 20 °C; **Black dashed line**: equilibrium *T*_g_ at 20 °C based on the kinetics model; **Green dashed line**: *T*_cure_ (20 °C).

In Figure 7, the *T*_g_ evolution of the 3M-3F630 vitrifying system is shown along with the *c_p_* measured in MTDSC and the heat flow measured in microcalorimetry (Thermal Activity Monitor, TAM) at 20 °C. The experimental *T*_g_s are obtained with separate measurements at an isothermal temperature of 20 °C for different reaction durations (before, during and after vitrification). The isothermal segments are followed by a cooling (at 20 K min^−1^) and heating step (at 5 K min^−1^) for the *T*_g_ determination in the heating step. For experiments where vitrification occurs, the heating was stopped just after the relaxation peak to limit further reaction at high temperature. Subsequently, the sample was rapidly cooled down and heated up to remeasure an iso-enthalpic *T*_g_. The change of conversion induced between the first heating and the second heating is simulated with the kinetics model to determine the conversion change Δ*x*. The correction applied to the *T*_g_, i.e., Δ*T*_g_, is then deduced from the *T*_g_–*x* relationship established in the previous subsection. The resulting experimental *T*_g_s are shown in Figure 7.

In order to correctly interpret the influence of vitrification on the measured *T*_g_s, it is crucial to simulate what should be the *T*_g_ evolution in case of a fully kinetically controlled cure at the same temperature. With the starting weight fractions and functionalities of the furan and maleimide compounds and a suitable set of kinetics parameters [40], the evolution of conversion at 20 °C is established and converted into *T*_g_ values using the *T*_g_–x relation. As can be seen in Figure 7, the *T*_g_ profile evolves towards an equilibrium value of 65 °C predicted by the kinetics model.

Before vitrification, the *c_p_* is nearly constant and the TAM exothermic heat flow intensity rapidly decreases (similar to the isothermal MTDSC measurements at different temperatures, see Section 3.1.1) while the *T*_g_ gradually increases with the Diels–Alder conversion according to the kinetics model prediction, as the Diels–Alder reactions take place in kinetically controlled conditions. As can be seen in Figure 7, the improved baseline quality of the TAM heat flow measurement allows to describe the ongoing reaction more accurately than in MTDSC.

When the region of the stepwise decrease Δ*c_p_* is entered, the crossing of the increasing experimental *T*_g_ with the temperature of cure at 20 °C is observed along with the start of the deviation of the increasing experimental *T*_g_ from the *T*_g_ profile predicted by the kinetics model. The latter is caused by vitrification which slows down the reaction because of the interference of segmental mobility restrictions. From that moment on, the reaction becomes diffusion-controlled and is no longer kinetically controlled. To correct for this effect in the simulation, a diffusion factor *DF* should be introduced, but this is out of the scope of this paper. Note that the coincidence between the drop of Δ*c_p_*, the crossing of experimental *T*_g_ and *T*_cure_ and the start of the deviation of the experimental *T*_g_ from the kinetics model prediction is dependent on the measurement conditions (see [38] for more details). After the start of vitrification, the reaction proceeds as shown by the still decreasing *c_p_* and TAM heat flow intensity and by the increasing experimental *T*_g_. However, the *T*_g_ increase noticeably slows down until reaching an apparent plateau around 44 °C, corresponding to 24 °C above the cure temperature of 20 °C and still 21 °C below the equilibrium *T*_g_ of 65 °C. This means that the conversion still increased by 0.26 after vitrification. The observation of the further progress of forward Diels–Alder reaction after vitrification is crucial for the self-healing capability in diffusion and mobility-controlled conditions as further demonstrated in Section 3.4.

### 3.3. De-Gelation

The concept of de-gelation is crucial in view of applications. The presence of a network ensures the structural integrity of the material and as such, it is important that the material keeps its network structure within the entire application temperature window. However, reversible systems such as Diels–Alder networks show a variation of conversion during changes of temperature. During heating, the conversion of a cured Diels–Alder network progressively decreases due to retro Diels–Alder reaction and eventually *x*_gel_ (or *x*_de-gel_) is reached. The reversibility of the network ensures the same critical conversion at gelation and de-gelation. The de-gelation temperature (*T*_de-gel_) should thus be determined in order to know to which temperature the material can be heated before the onset of viscous flow.

**Figure 8:**
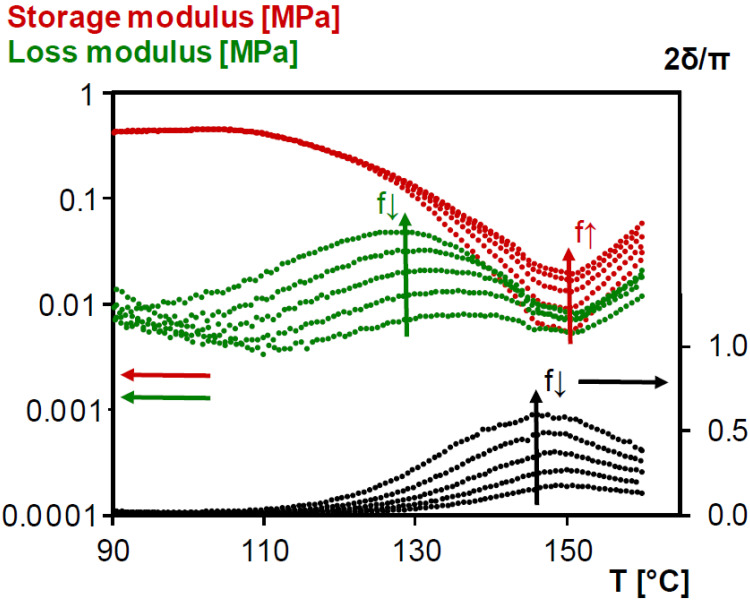
Storage modulus, loss modulus and normalized loss angle of dynamic rheometry during heating at 0.5 K min^−1^ between 90 and 160 °C of previously cured reversible network 3M-3F630. **Black**: normalized loss angle; **Red**: storage modulus; **Green**: loss modulus.

As described in Section 3.1.2., dynamic rheometry measurements are suitable to characterize gelation, but they should also be suitable for de-gelation measurements. Figure 8 shows the evolution of rheometrical loss angle *δ*, storage modulus G′ and loss modulus G″ of thermally cured 3M-3F630 reversible network after an isothermal segment for 15 min at 90 °C before being heated at 0.5 K min^−1^ up to 160 °C. The same information is given for the photo-cured reversible network 3M-F375PMA in Figure S4 (in the Supplementary Materials). The isothermal segment at 90 °C is necessary to stabilize the materials above their *T*_g_ to avoid any interference of the vitrification process. For de-gelation the same criterion as for gelation, namely the frequency-independency of the loss angle, can clearly not be used. For the 3M-3F630 system, from values close to 0° the loss angles start to increase around 120 °C to reach a maximum before 150 °C and afterwards going down again without crossing. The increase of loss angle could be the start of the de-gelation process, but the expected value should rise to 60°–65° (see Section 3.1.2.) which is not the case even at the lowest frequency. Another experimental criterion to define gelation (and de-gelation), i.e., a phase angle of 45° giving equal elastic and viscous contributions G′ = G″ and corresponding to 2*δ*/*π* = 0.5 in Figure 8 [50,51,52,53], does not allow an unambiguous determination of *T*_de-gel_ either. The observed decrease of loss angle above 150 °C (by an increase of the storage modulus, see Figure 8) is probably due to side reactions at these high temperatures, such as the radical homopolymerization of maleimide groups giving extra (irreversible) crosslinking [39,57]. An alternative approach to determine de-gelation is by means of the critical gel conversion *x*_gel_ = 0.50 (see Section 3.1.2) which is also equal to *x*_de-gel_. At a heating rate of 0.5 K min^−1^ starting from 90°C, *T*_de-gel_ can be simulated using the kinetic parameters of [40], leading to a value of 156 °C. It confirms that the rheometrical measurement of de-gelation is masked by side-reactions at the high temperature side, but probably lies beyond 150°C so that structural integrity is certainly maintained up to these temperatures.

For the 3M-F375PMA system, the loss angles start to slightly increase around 130 up to 160 °C but stays far below 45° (2*δ*/*π* = 0.5) without crossing (see Figure S4). This shows that the 3M-F375PMA system does not de-gel up to 160 °C, which is also well above the usual application temperatures such as for photovoltaic encapsulants.

### 3.4. Proof of Self-Healing of the Reversible Network 3M-F375PMA at Ambient Temperature

#### 3.4.1. Reversibility of the Poly(methacrylate) Network 3M-F375PMA

The network 3M-F375PMA is synthesized according to a two-step procedure described in detail in Section 2.2 Synthesis. Transmission FTIR spectroscopy is used to study the reversibility of the synthesized 3M-F375PMA in a KBr pellet. Diels–Alder bonds that are present in the polymer network can undergo retro Diels–Alder reaction to form maleimide and furan groups. In FTIR spectroscopy, this can be recognized by the increasing peak intensity related to the maleimide ring deformation at 695 cm^−1^.

**Figure 9:**
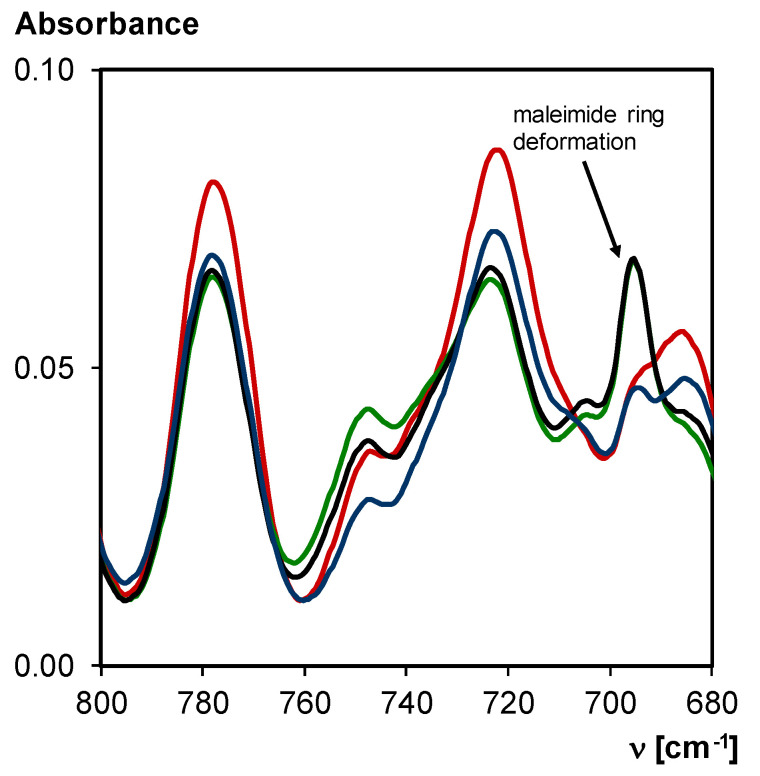
Transmission FTIR spectra of 3M-F375PMA in a KBr pellet at 20 °C, normalized to the C–O–C symmetric stretching vibration at 1200–1050 cm^−1^ (not shown). **Red**: material at starting conditions; **Green**: 1st time retro Diels–Alder reaction (after 15 min at 100 °C); **Black**: 4th time retro Diels–Alder reaction (after 15 min at 100 °C); **Blue**: material at starting conditions after thermal cycling (fourth cycle after seven days at 20 °C).

First, a FTIR spectrum of 3M-F375PMA is taken at 20 °C. This spectrum represents the starting conditions of the material with high Diels–Alder conversion. Then, the sample is heated to 100 °C for 15 min, reducing the Diels–Alder conversion through retro Diels–Alder reaction. The sample is then quickly cooled down to 20 °C, and FTIR analysis is performed again, showing an increased maleimide concentration. Subsequently, the material is left at 20 °C for seven days, and then another FTIR spectrum is taken, which resembles the initial FTIR spectrum of the material in starting conditions. Furthermore, it can be recognized from the decreased maleimide ring deformation peak that Diels–Alder bonds were formed again. This procedure is repeated multiple times in order to show that 3M-F375PMA reversibly undergoes Diels–Alder and retro Diels–Alder reaction for at least four thermal cycles. Figure 9 shows an overlay of the FTIR spectra between 800 and 680 cm^−1^.

**Figure 10:**
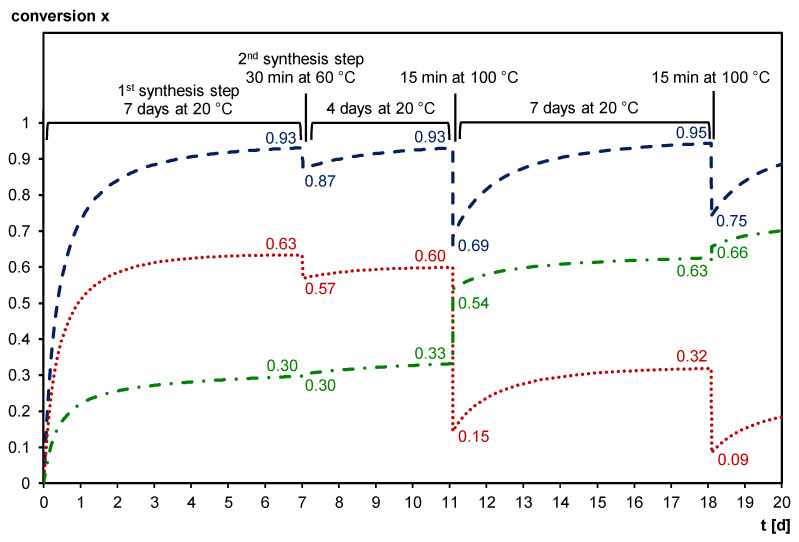
Simulated evolution of the Diels–Alder conversion for the formation of 3M-F375MA (first synthesis step, seven days at 20 °C), the subsequent photopolymerization to form 3M-F375PMA (second synthesis step, 30 min at 60 °C), a recovery period of four days at 20°C, the first heating to 100 °C for 15 min, a recovery period for seven days at 20 °C, and the second heating to 100 °C for 15 min. **Dashed blue line**: total Diels–Alder conversion *x* (= *x_endo_* + *x_exo_*); **Dotted red line**: *endo* Diels–Alder adduct conversion *x_endo_*; **Dash-dotted green line**: *exo* Diels–Alder adduct conversion *x_exo_*.

Figure 10 shows the simulated Diels–Alder conversion and the influence of thermal treatment on the Diels–Alder conversion, using the kinetic parameters of [40]. However, as mentioned above, this kinetic model only fully applies to kinetically controlled reactions, and a diffusion factor *DF* should be introduced to correctly describe reactions in diffusion-controlled conditions. During the first step of the synthesis, i.e., the formation of Diels–Alder bonds to yield the prepolymer 3M-F375MA, the total Diels–Alder conversion *x* increases from 0 to 0.93. During the second step of the synthesis, i.e., UV-polymerization of the methacrylate bonds at 60 °C to form 3M-F375PMA, *x* is slightly reduced to 0.87. However, after four days at 20 °C, the initial *x* of 0.93 is recovered. As discussed, FTIR spectroscopy is used to demonstrate the reversibility of the Diels–Alder reaction. The simulation in Figure 10 shows that through heating the sample to 100 °C for 15 min, the total Diels–Alder conversion is reduced to 0.69. During a subsequent recovery step, i.e., seven days at 20 °C, the initial total Diels–Alder conversion is restored, and even slightly increased to 0.95. Repeated heating to 100 °C for 15 min reduces the total Diels–Alder conversion again, in this case to 0.75. Thus, the simulation confirms the findings from the FTIR experiments. Note that while the total Diels–Alder conversion is reaching almost the same level after each long recovery step at 20 °C, the *exo*/*endo* ratio is changing in favor of the more stable *exo* cycloadduct each time a temperature increase is involved in the temperature cycle.

**Figure 11:**
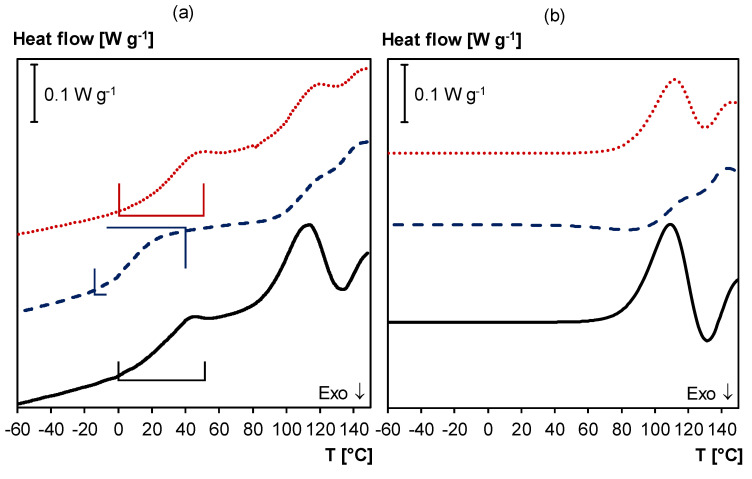
(**a**) DSC thermogram of 3M-F375PMA. **Full black line**: first heating; **Dashed blue line**: second heating (immediately after first heating); **Dotted red line**: third heating (after seven days at 20 °C). Frames indicate *T*_g_ region. (**b**) Simulated normalized heat flows of first, second and third heating (without *T*_g_), showing effect of ongoing Diels–Alder reactions, using the kinetic parameters of [40]. Curves vertically shifted for clarity.

Moreover, DSC (heating/cooling at 10 K min^−1^) is used to study thermal reversibility of the 3M-F375PMA network (Figure 11a). The DSC thermogram of the sample in starting conditions (full black curve in Figure 11a) shows a broad glass transition temperature between 0 and 55 °C and two endothermic reaction peaks between 70 and 150 °C due to the retro Diels–Alder reaction of the *endo* and *exo* Diels–Alder adducts, respectively. If the same sample is subjected to a second DSC experiment immediately after the first measurement (dashed blue curve in Figure 11a), the temperature range of the glass transition is shifted to lower temperatures (between –15 and 40 °C), and the endothermic retro Diels–Alder reaction enthalpy is reduced. Both phenomena are caused by the fact that the material’s crosslink density is reduced compared to its starting conditions because the sample was not allowed enough time to recover between the first and the second heating. However, if the material is allowed enough time to recover between DSC experiments (e.g., seven days at 20 °C), it can return to its starting equilibrium, and the same *T*_g_ and retro Diels–Alder peaks as in the first heating are detected (dotted red curve in Figure 11a). Thus, the DSC experiments confirm that 3M-F375PMA is reversible under these conditions. When comparing the endothermic reaction peaks of the first and the third DSC experiment in Figure 11a, it becomes apparent that the two thermograms do not have the exact same shape between 70 °C and 150 °C. As mentioned earlier, *endo* and *exo* stereoisomers of the Diels–Alder adduct are formed during Diels–Alder reaction between maleimide and furan. The *endo* stereoisomer is kinetically favored, while the *exo* stereoisomer is thermodynamically more stable [38], and the ratio between these two stereoisomers influences the shape of the endothermic and exothermic reaction peaks in the DSC experiments.

Figure 11b shows the simulated normalized heat flow when applying the same temperature profile as in the DSC experiments, using the kinetic parameters of [40], which is in good agreement with the experimental results. Note that the simulation does not include the *T*_g_s since changes in the *c_p_* are not simulated.

#### 3.4.2. Structural Integrity and Mechanical Robustness of the Reversible Poly(methacrylate) Network 3M-F375PMA

**Figure 12:**
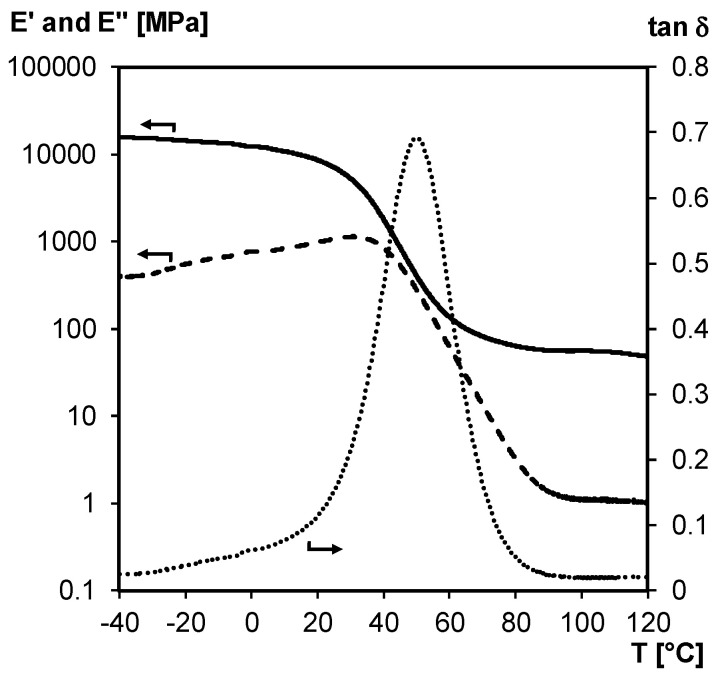
DMA of 3M-F375PMA network film measured in tension mode. Storage modulus *E’* (**full line**), loss modulus *E’’* (**dashed line**) and tan *δ* (**dotted line**).

The structural integrity of 3M-F375PMA network film is studied between –40 and 120 °C via non-isothermal DMA experiments in tension mode (Figure 12). According to the loss modulus *E’’* and tan *δ* curves, which show one broad *T*_g_ centered around 50 °C (peak maximum of tan *δ*), the material can be considered a homogeneous thermoset network at ambient temperature. This is also in agreement with the DSC thermograms of Figure 11a. Additionally, the storage modulus *E’* reaches a rubbery plateau at temperatures above the *T*_g_ (around 50 MPa measured up to 120 °C), meaning that the poly(methacrylate) network maintains its structural integrity over a broad temperature range. Here, acceptable mechanical properties are ensured by the irreversible poly(methacrylate) chains even if the material’s crosslink density is reduced through retro Diels–Alder reaction.

#### 3.4.3. Self-Healing of 3M-F375PMA Powder Rectangular Bar Samples at Ambient Temperature

**Figure 13:**
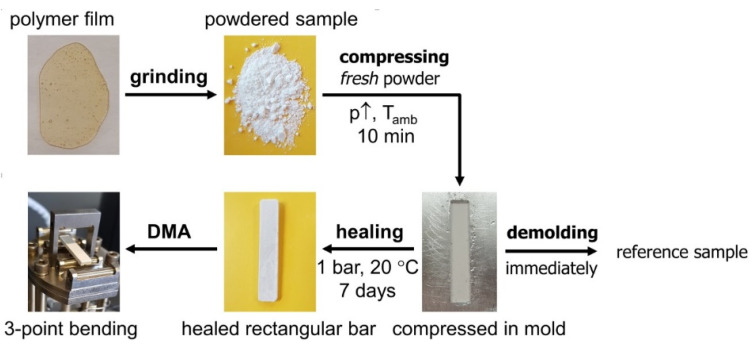
Self-healing of 3M-F375PMA at 20 °C. A polymer film is ground into fine powder, compressed in a metal mold at elevated pressure at *T*_amb_ for 10 min, demolded after self-healing for seven days at 1 bar and 20 °C, and analyzed by DMA in 3-point bending mode. A reference is prepared by immediate demolding after compression.

The ambient-temperature self-healing capability of the poly(methacrylate) network 3M-F375PMA regarding microcracks is tested in the (partially) vitrified state, following a specific procedure [38,39], as illustrated in Figure 13. First, a film of 3M-F375PMA is ground into a fine powder. Here, the Diels–Alder bonds formed in the reaction between maleimide and furan (indicated with red arrows in Figure 1) are preferentially broken, because they are weaker than other carbon-carbon σ-bonds [34,36]. Second, the fresh powder is compressed for 10 min at ambient temperature in a mold with the required dimensions. This way, the powder particles are brought into close contact with each other, mimicking microcracks. Third, the compressed sample is allowed to self-heal for seven days at 20 °C and 1 bar. Here, Diels–Alder bonds are formed between the surfaces of the powder particles, restoring the Diels–Alder equilibrium and leading to consolidation of the powder rectangular bar. Finally, the sample is demolded and analyzed by means of DMA in 3-point bending mode. A reference sample is prepared by omitting the healing step and immediately demolding the compressed powder sample, which, however, cannot be analyzed by means of DMA, because no bulk sample is obtained.

**Figure 14:**
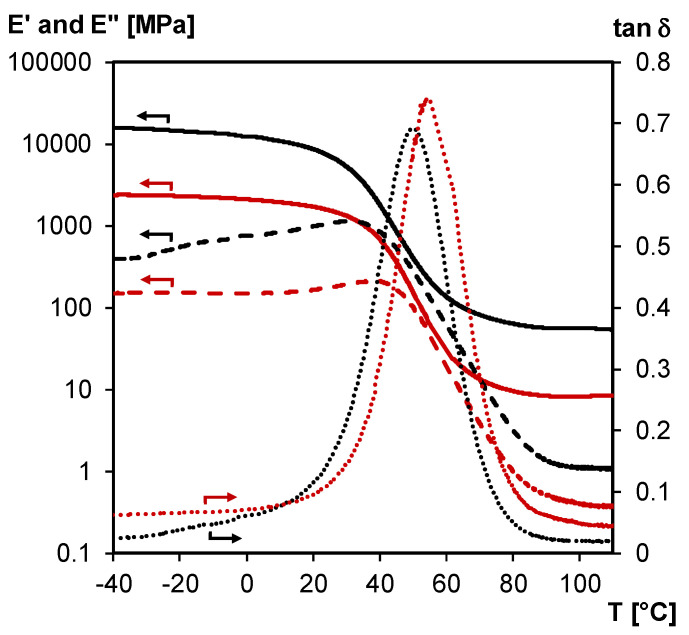
DMA of the 3M-F375PMA, measured on film in tension mode (undamaged, **black**) and on powder rectangular bar in 3-point bending mode (healed at 20 °C after damage, **red**). Storage modulus *E’* (**full lines**), loss modulus *E’’* (**dashed lines**) and tan *δ* (**dotted lines**).

Figure 14 shows an overlay of the DMA results obtained for an undamaged 3M-F375PMA film (measured in tension mode) and a self-healed rectangular bar sample prepared from fresh 3M-F375PMA powder. The results show that the mechanical properties of the damaged material are (partially) restored during the healing step at 20 °C, and that a powder rectangular bar with acceptable mechanical properties, around 1.7 GPa at 20 °C, is obtained. However, the initial mechanical properties of the undamaged sample are not fully recovered, probably by remaining voids in the self-healed bar of compressed powder and also due to the difference in measuring mode (bending against tension). Furthermore, the *T*_g_ of the self-healed rectangular bar is slightly shifted to higher temperatures (i.e., by about 5 °C, at temperature of maximum tan *δ*) compared to the *T*_g_ of the undamaged film sample. Note that no healing takes place in the same conditions if *aged* powder is used of which the surface area has a restored Diels–Alder equilibrium before compression in the mold.

**Figure 15:**
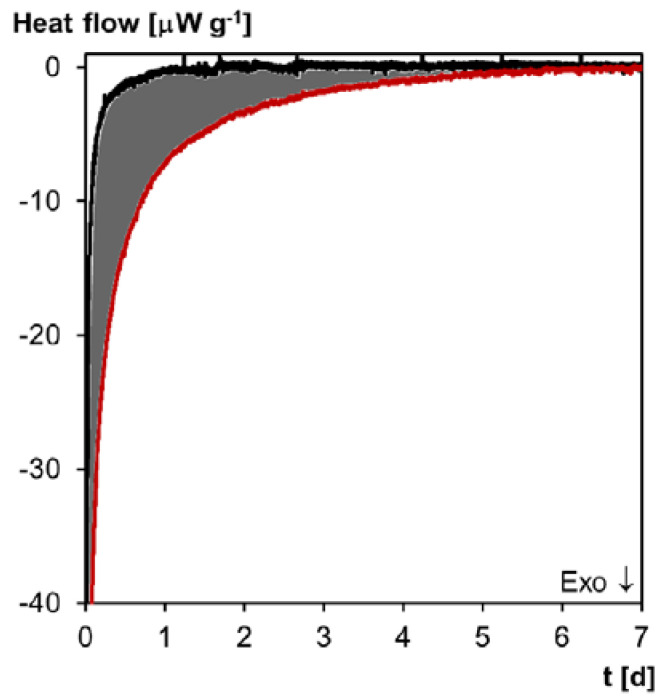
Microcalorimetry of 3M-F375PMA. Normalized heat flow as a function of time at 20 °C. **Black line**: undamaged film sample; **Red line**: compressed fresh powder; **Grey area**: Diels–Alder reaction enthalpy of fresh powder.

As discussed above, during the healing step, Diels–Alder bonds are reformed in freshly ground 3M-F375PMA powder in order to restore Diels–Alder equilibrium. The heat flow originating from this reaction can be measured by means of isothermal microcalorimetry. Figure 15 shows the normalized heat flow of freshly ground 3M-F375PMA powder after 10 min compression at ambient temperature. Here, the residual heat flow of undamaged 3M-F375PMA film, which was stored for three weeks at ambient temperature prior to the experiment, serves as reference. Both measurements are performed over seven days at 20 °C, i.e., under the same conditions as the healing step during the preparation of powder rectangular bar samples. The net exothermic heat flow difference, Δ*H_r_* = −2.16 J g^−1^, which is determined by integration of the area between the two curves (shaded area in Figure 15), verifies that Diels–Alder reaction takes place in fresh 3M-F375PMA powder. Thus, it is confirmed that Diels–Alder reaction is responsible for the material’s self-healing properties through the formation of Diels–Alder bonds between powder particle surfaces.

### 3.5. Ambient-Temperature Healing Application: Encapsulant in Photovoltaics

The results of Section 3.4 show that self-healing of the poly(methacrylate) network 3M-F375PMA is feasible in (partially) vitrified conditions at ambient temperature, which can be valuable in outside applications, such as in self-healing encapsulant layers in photovoltaics. In photovoltaic modules, solar cells convert solar radiation into electricity. Therefore, overlying layers are required to be highly transparent for ultraviolet and visible light. Preliminary tests on 3M-F375PMA film with a thickness of 0.50 mm show a transmittance of T > 85% between 1000 and 515 nm, and a UV-cutoff at 315 nm (Figure S5 in the Supplementary Materials), which is comparable to poly(ethylene-co-vinyl acetate) (EVA) used as photovoltaic encapsulants [58].

**Figure 16:**
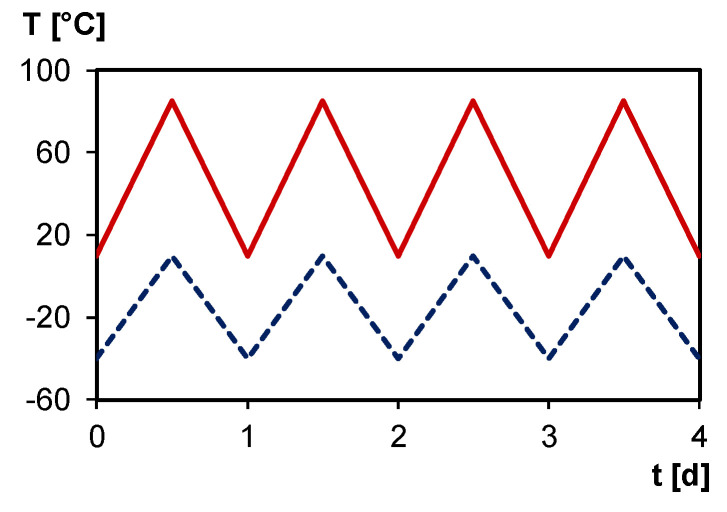
Typical daily temperature cycle in summer (**full red line**) and in winter (**dashed blue line**).

Typically, photovoltaic modules are exposed to operating temperatures between –40 and 85 °C, and, therefore, a typical daily temperature cycle (Figure 16) enables self-healing in ambient conditions using solar energy as trigger.

Photovoltaic modules are solid composite structures, comprised of solar cells, glass front sheet, backsheet and two layers of encapsulant. Due to mismatching thermal expansion coefficients of the different layers, thermal cycling can lead to thermal stress [59]. Therefore, conventional encapsulant layers, such as EVA and polyolefin elastomer (POE) [58], are susceptible to the formation of micro-defects, which may impair solar panel efficiency if they grow into larger defects. Introducing self-healing properties into the encapsulant material can help prevent crack growth in the encapsulant layer through regular healing of newly formed micro-defects.

**Figure 17:**
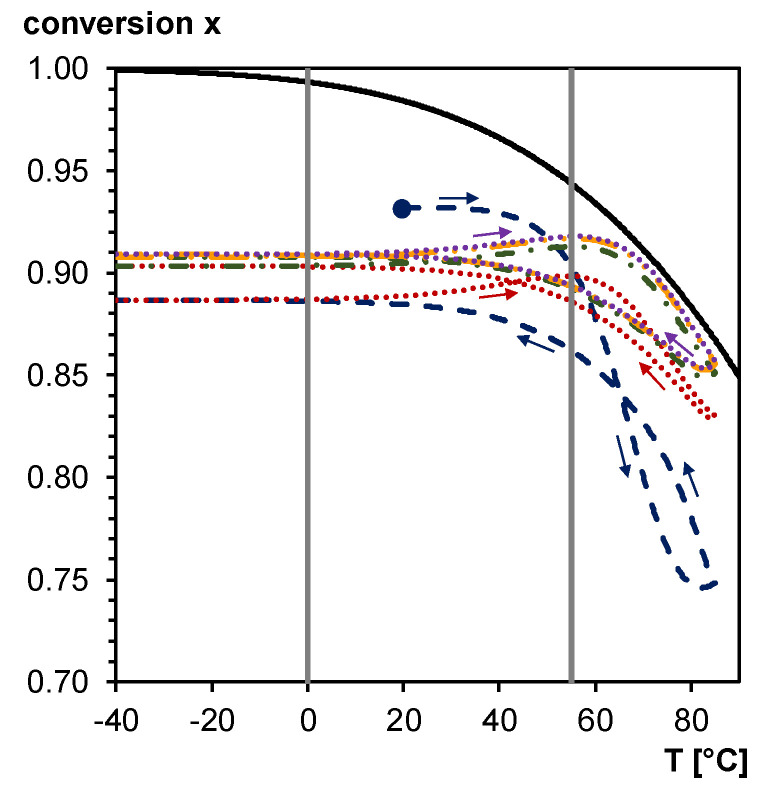
Equilibrium conversion (**full black line**) and simulated Diels–Alder conversion for the 3M-F375PMA reversible network in five consecutive day cycles. **Dashed blue line**: first day cycle (starting from the simulated Diels–Alder conversion after the two-step synthesis of 0.93 at 20°C); **Dotted red line**: second day cycle; **Dash-dotted green line**: third day cycle; **Dash-dot-dotted orange line**: fourth day cycle; **Dotted purple line**: fifth day cycle. Equilibrium hysteresis loop is reached after five day cycles. **Vertical grey lines**: *T*_g_ region of the 3M-F375PMA reversible network.

The daily temperature cycling causes a continuous change in Diels–Alder conversion, and, therefore, can be exploited as thermal trigger for self-healing. Figure 17 shows the simulated Diels–Alder conversion between –40 and 85 °C for a typical heating/cooling rate during a day cycle, using the kinetic parameters of [40]. Simulations are done starting from the simulated Diels–Alder conversion of 0.93 after the two-step synthesis at 20 °C (see Figure 10), and without accounting for diffusion limitations (full kinetic control). The equilibrium conversion line is indicated for comparison (full black line in Figure 17). At heating rates comparable with these of a daily cycle (e.g., 0.17 K min^−1^ in case of cycling between −40 and 85 °C in 12 h) deviations from the equilibrium conversion are observed as a result of too slow reaction kinetics [34]. At the low temperature side of the thermal cycle, the Diels–Alder conversion stays nearly constant as if dictated by diffusion control and mobility restrictions below and inside the broad glass transition region of the 3M-F375PMA reversible network (indicated by vertical grey lines between 0 and 55 °C in Figure 17). At the higher temperature side of the thermal cycle, almost coinciding with temperatures above the upper limit of the glass transition region, the increasing reaction kinetics give clear variations of the Diels–Alder conversion. After a few transient day cycles the equilibrium curve is approached at the high temperature side, and an equilibrium hysteresis loop is obtained. This simulation proves that the 3M-F375PMA reversible network can be used as an encapsulant for photovoltaics with self-healing capacity in a broad ambient temperature window, typically between −40 and 85 °C. Moreover, the fact that the Diels–Alder conversion never decreases below 0.75 (first transient cycle) or below 0.85 (consecutive cycles and equilibrium hysteresis loop) ensures mechanical robustness of the protective encapsulant in these outside application conditions. Note that the conversions of the simulated day cycles will vary with the specified experimental conditions, such as exact heating and cooling rates and concentrations of the furan and maleimide functionalities in the synthesized reversible network. However, the main conclusions stay valid.

## 4. Conclusions

Two reversible polymer networks, 3M-3F630 and 3M-F375PMA, were synthesized and studied in terms of network formation and potential self-repair in diffusion-controlled conditions. Information on these aspects is scarce in the literature of self-healing polymer materials.

The progress of the Diels–Alder reactions was successfully followed for the 3M-3F630 system. (Partial) vitrification could be observed with MTDSC, while network formation was observed by rheometrical measurements. At 20 °C, clear progress of the reaction in the vitrified state was still observed as the *T*_g_ of the material increased 24 °C above the cure temperature, which gives a straightforward indication of the self-repair potential of such systems in vitrified conditions.

In view of applications of self-healing polymer materials in general, the persistence of a network structure is primordial to conserve the structural/geometrical integrity of the material, also at more elevated temperatures during the sealing step. The structural integrity of the 3M-3F630 and 3M-F375PMA networks was guaranteed to at least 150 °C. Moreover, mechanical robustness in 3M-F375PMA was maintained to at least 120 °C by the poly(methacrylate) chains, which are the result of the UV-polymerization step of the Diels–Alder pre-polymer 3M-F375MA.

The thermal reversibility of 3M-F375PMA was proven to be repeatable if enough time is given at the low-temperature side of the thermal cycle to recover the initial state.

The ability to self-repair microcracks in the vitrified state at 20 °C was demonstrated.

In addition, the potential of the 3M-F375PMA system as an encapsulant in photovoltaics was explored. Kinetic simulations showed that a continuous change of Diels–Alder conversion is observed when the material is subjected to daily temperature cycles between −40 and 85 °C. This shows that the temperature cycle could act as a trigger for the self-healing of defects in the protective coating. The broad *T*_g_ of the network between 0 and 55 °C and the diffusion-controlled Diels–Alder reaction inside and below this *T*_g_ region is hardly influencing the change of conversion during thermal cycling.

As a final remark, the importance of kinetic simulations based on Diels–Alder equilibrium reactions of *endo* and *exo* cycloadducts and the essential role of reliable kinetics to interpret the experimental results should be emphasized. Examples are: (i) the proof of diffusion-controlled progress of Diels–Alder reactions during vitrification, (ii) the effect of thermal cycling on the total Diels–Alder conversion and on the changing *exo*/*endo* ratio in favor of the more stable *exo* cycloadduct each time a temperature increase is involved, (iii) the indirect calculation of the de-gelation temperature which is not directly measurable because of side reactions at higher temperatures, (iv) the variations during day cycles of the transients and equilibrium hysteresis loops in Diels–Alder conversion with changes in heating/cooling rates and furan and maleimide concentrations in the self-healing encapsulants for photovoltaics.

## Supplementary Materials

Available online at https://www.mdpi.com/2073-4360/12/11/2543/s1. Table S1. Kinetic and thermodynamic parameters from [40]; Figure S1. Additional chemical structures of the starting reagents used in Figure S2 to cure model reversible network M400-3F251; Figure S2. Microcalorimetry and simulated evolution of the normalized heat flow as a function of time at 20 °C of the M400-3F251 model reversible network prepared the same way as the reversible thermosetting network 3M-3F630 (See 2.2 Synthesis of the main text); Figure S3. MTDSC and microcalorimetry of 3M-F375PMA at 20 °C; Figure S4. Storage modulus, loss modulus and normalized loss angle of dynamic rheometry during non-isothermal treatment at 0.5 K min^-1^ between 90 and 160 °C of previously photo-cured reversible network 3M-F375PMA; Figure S5. Transmittance determined by means of UV-Vis spectroscopy for a 500 μm thick 3MF375PMA film.
