# Supplementary material for: Self-Healing in Mobility-Restricted Conditions Maintaining Mechanical Robustness: Furan–Maleimide Diels–Alder Cycloadditions in Polymer Networks for Ambient Applications
Source: Polymers (Basel). 2020 Oct 30;12(11):2543. doi: 10.3390/polym12112543 (PMC7692822; doi:10.3390/polym12112543)
Supplement: Supplementary file 1 [file polymers-12-02543-s001.pdf]

Supplementary Material

Self-Healing in Mobility-Restricted Conditions Maintaining Mechanical Robustness: Furan-Maleimide Diels-Alder Cycloadditions in Polymer Networks for Ambient Applications

[1] Physical Chemistry and Polymer Science (FYSC), Vrije Universiteit Brussel (VUB), Pleinlaan 2, 1050 Brussels, Belgium, Dorothee.Ehrhardt@vub.be (D.E.); Kurt.Durme-van@dsm.com (K.V.D.); Bruno.Van.Mele@vub.be (B.V.M.); Niko.Van.den.Brande@vub.be (N.V.d.B.)
[2] Organic Chemistry (ORGC), Vrije Universiteit Brussel (VUB), Pleinlaan 2, 1050 Brussels, Belgium, Jolien.Bertouille@vub.be (J.B.)
[3] DSM Advanced Solar, Urmonderbaan 22, 6167 RD Geleen, the Netherlands
† Shared first authorship
* Correspondence: Jessica.Mangialetto@vub.be (J.M)

Table S1 Kinetic and thermodynamic parameters from [40].

Kinetic/thermodynamic parameters	Parameter values
$\ln(A_{DA,endo})$ (kg mol^{-1} s^{-1})	13.4
$E_{DA,endo}$ (kJ mol^{-1})	59.4
$\ln(k_{DA,endo})$ (s^{-1}) at 298 K	-10.5
$\ln(A_{rDA,endo})$ (s^{-1})	30.9
$E_{rDA,endo}$ (kJ mol^{-1})	113.1
$\ln(k_{rDA,endo})$ (s^{-1}) at 298 K	-14.7
$\ln(A_{DA,exo})$ (kg mol^{-1} s^{-1})	14.7
$E_{DA,exo}$ (kJ mol^{-1})	64.6
$\ln(k_{DA,exo})$ (s^{-1}) at 298 K	-11.4
$\ln(A_{rDA,exo})$ (s^{-1})	31.4
$E_{rDA,exo}$ (kJ mol^{-1})	123.6
$\ln(k_{rDA,exo})$ (s^{-1}) at 298 K	-18.4
$\Delta_r H^0_{endo}$ (kJ mol^{-1})	-53.7
$\Delta_r S^0_{endo}$ (J mol^{-1} K^{-1})	-145.1
$\Delta_r H^0_{exo}$ (kJ mol^{-1})	-59.0
$\Delta_r S^0_{exo}$ (J mol^{-1} K^{-1})	-138.9

The data of Table S1 are based on furan-maleimide DA reactions where secondary H-bonding interactions by OH-functionalities linked to the furan compounds are involved. They are suitable for the simulation of the cure kinetics of the two thermosetting networks of this paper, for which secondary H-bonding interactions by NH-functionalities linked to the furan compounds are involved.

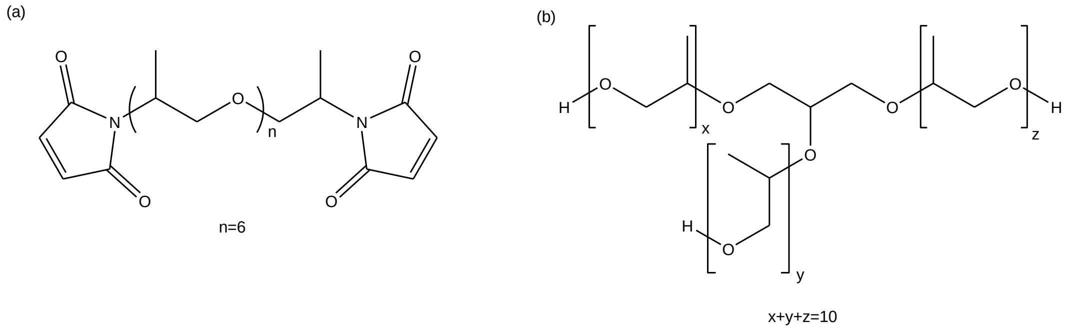

Figure S1. Additional chemical structures of the starting reagents used in Figure S2 to cure model reversible network M400-3F251: (a) amorphous bismaleimide M400 (M400, M_{eq} = 308 g mol^{-1} , f=1.95, Specific Polymers); (b) glycerol-based polyol Daltolac R251 (M = 673 g mol^{-1}, Huntsman).

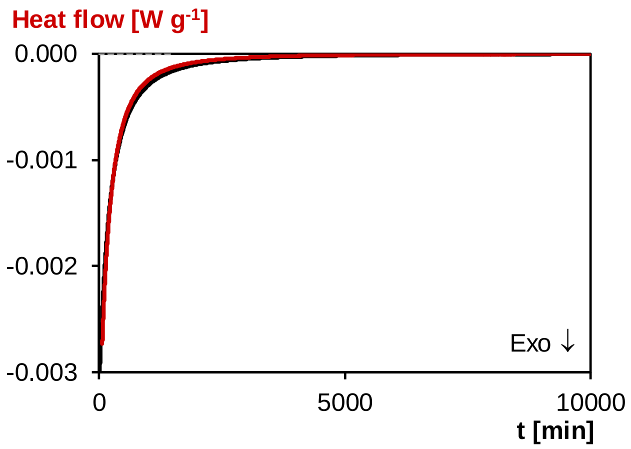

Figure S2. Microcalorimetry and simulated evolution of the normalized heat flow as a function of time at 20 °C of the M400-3F251 model reversible network prepared the same way as the reversible thermosetting network 3M-3F630 (See 2.2 Synthesis of the main text). **Black**: simulated normalized heat flow using data set of Table S1; **Red**: experimental normalized heat flow.

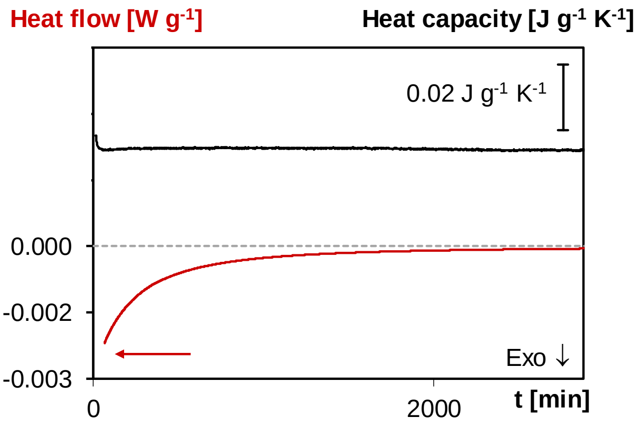

Figure S3. MTDSC and microcalorimetry of 3M-F375PMA at 20 °C. **Black**: (specific) heat capacity as a function of time measured in MTDSC; **Red**: normalized heat flow as a function of time measured in TAM.

The normalized heat flow intensity at the start of the reaction is around 3 times smaller than for 3M-3F630 in the same reaction conditions and the decrease of exothermicity over time is much slower, showing the effect of the concentration on the reaction rate which for 3M-F375MA is about 1.7 times smaller than for 3M-3F630. The c_p evolution of the 3M-F375MA system clearly presents no sudden Δc_p drop typical of vitrification, which shows that the system does not enter a vitrified state during cure at 20 °C.

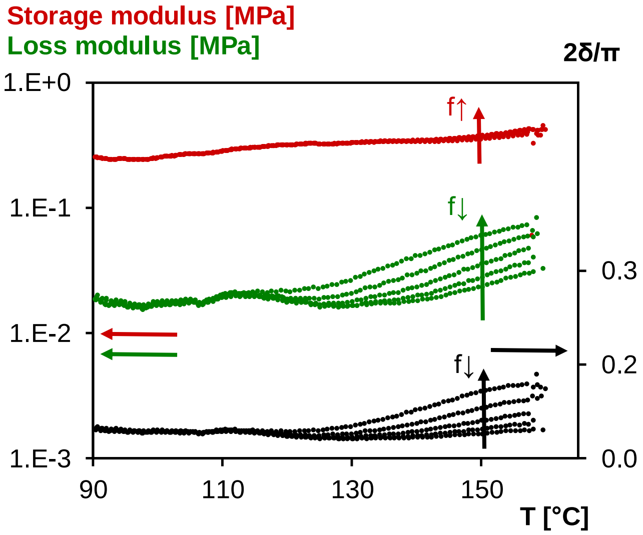

Figure S4. Storage modulus, loss modulus and normalized loss angle of dynamic rheometry during non-isothermal treatment at 0.5 K min^{-1} between 90 °C and 160 °C of previously photo-cured reversible network 3M-F375PMA. **Black**: normalized loss angle; **Red**: storage modulus; **Green**: loss modulus.

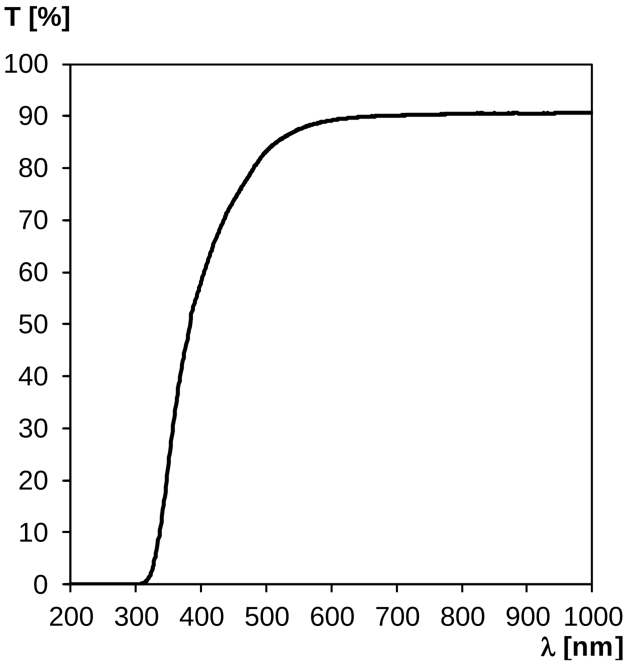

Figure S5. Transmittance determined by means of UV-Vis spectroscopy for a 500 μm thick 3M-F375PMA film. 1000-515 nm: T > 85%, 515-435 nm: T > 70%, UV-cutoff: 315 nm.
